# Supplementary material for: Multi-faceted plasmonic nanocavities
Source: Nanophotonics. 2023 Oct 4;12(20):3931–44. doi: 10.1515/nanoph-2023-0392 (PMC11501932; doi:10.1515/nanoph-2023-0392)
Supplement: Supplementary file 1 — Supplementary Material Details [file j_nanoph-2023-0392_suppl_001.pdf]

Supplementary Information—Multi-faceted plasmonic nanocavities

Kalun Bedingfield[1], Eoin Elliott[1,2], Arsenios Gisdakis[1], Nuttawut Kongsuwan[3,4], Jeremy J Baumberg[*2], and Angela Demetriadou[†1]

[1]*School of Physics and Astronomy, University of Birmingham, Edgbaston, Birmingham, B15 2TT, UK*

[2]*NanoPhotonics Centre, Cavendish Laboratory, University of Cambridge, Cambridge CB3 0HE, UK*

[3]*Thailand Center of Excellence in Physics, Ministry of Higher Education, Science, Research and Innovation, Bangkok 10400, Thailand*

[4]*Quantum Technology Foundation (Thailand), Bangkok 10110, Thailand*

Dated: August 14, 2023

1 Higher Order Quasi-Normal Modes

To extend on the first six modes of the TSoM geometry shown in the main manuscript, here the near-field charge distribution of the next most energetically favourable mode is shown in Figure S1A. This is the $(3, 3)$ mode, which exhibits the expected $2m$-lobed distribution. In

*jjb12@cam.ac.uk
†a.demetriadou@bham.ac.uk

the corresponding far-field emission profile of Figure S1B, the same $2m$-lobed distribution is observed—contrary to that identified in previous works ([1] and [2]). This minor correction arises due to the incredibly fine meshing required to obtain the far-field emissions using RETOP [3]. Although we have more accurately identified this far-field emission profile, it is in general too computationally expensive to distinguish the lobes of modes with $m \geq 3$ in the far-field.

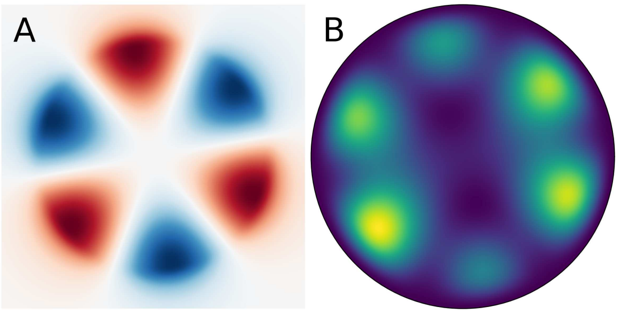

Figure S1: Quasi-normal $(3, 3)$ mode of the circularly faceted TSoM geometry. (A) Near-field charge distribution of the normalised QNM electric field ($\mathrm{Re}[E_{z,lm}]$)—on the xy-plane through the centre of the cavity. (B) Far-field emission pattern of the normalised time averaged Poynting flux $\langle S_{lm} \rangle$.

2 Edge Rounding

For a realistic representation of the polyhedral nanoantenna geometries, a curvature to the edges of each structure is applied. This rounding is schematically shown in Figure S2, whereby we can define a single quantity ρ that describes the rounding of the edges.

This section is dedicated to unveiling some of the subtleties involving the edge rounding in these polyhedral nanoantenna systems, and the strong impact this can have on the QNMs in both the near- and far-fields. One mode that is particularly sensitive to the rounding of the edges is the second $(2, 2)$ mode of the RhoM-Sq22 geometry—whose anti-nodes align with the facet edges. Figure S3 compares the near-field charge distributions and far-field emission profiles for this facet with edge rounding of $\rho = 1$nm (top) and $\rho = 5$nm (bottom). As the

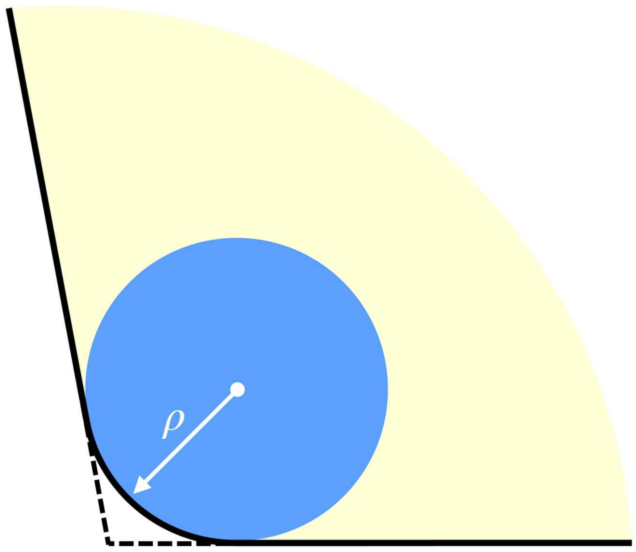

Figure S2: Schematic showing the edge rounding, ρ, applied to each of the polyhedral geometries.

rounding is increased, the effective elongation of the facet is more pronounced for the second $(2, 2)$ mode and the $(2, 0)$ mode—whilst leaving the other modes largely unchanged. In the near-field, the $(2, 0)$ mode is visibly stretched along the elongation axis, and the lost central node of the $(2, 2)$ mode becomes more significantly non-zero. In the far-field this non-zero centre fields leads to the $(2, 2)$ mode emitting just like an $m = 0$ mode.

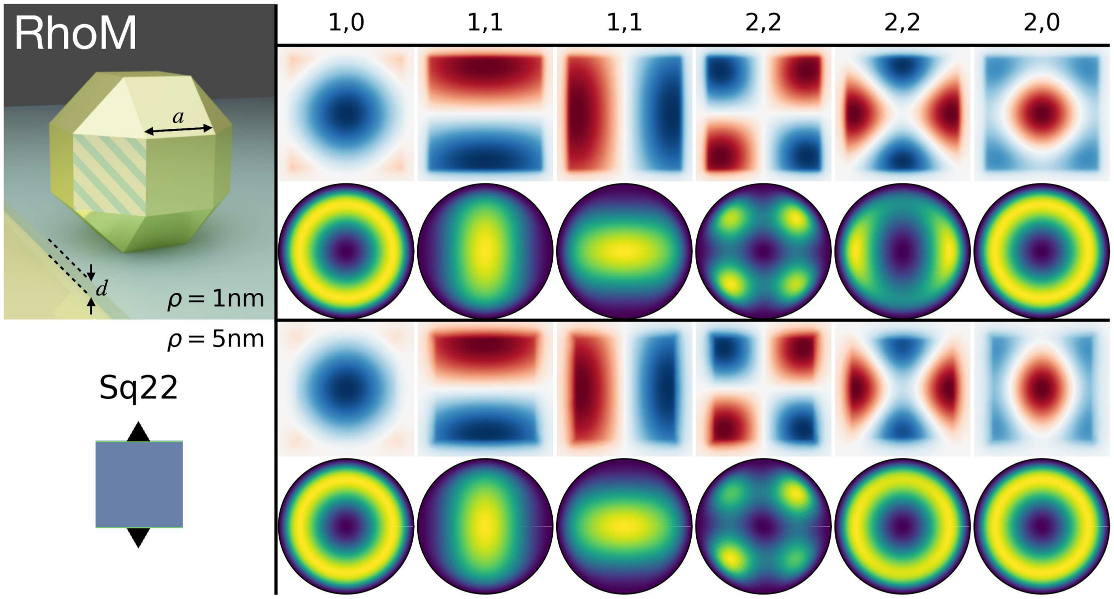

Figure S3: QNMs of the RhoM geometry assembled on it's Sq22 facet for a characteristic edge length a and spacer thickness d, and edge rounding of (rows 1 and 2) $\rho = 1$nm and (rows 3 and 4) $\rho = 5$nm. Rows 1 and 3 are the normalised QNM electric near-fields ($\mathrm{Re}[E_{z,lm}]$) on the xy-plane through the centre of the cavity. Rows 2 and 4 are the normalised time averaged far-field Poynting flux $\langle S_{lm} \rangle$. The modes from left to right are: $(1, 0)$, $(1, 1)$, $(1, 1)$, $(2, 2)$, $(2, 2)$, $(2, 0)$.

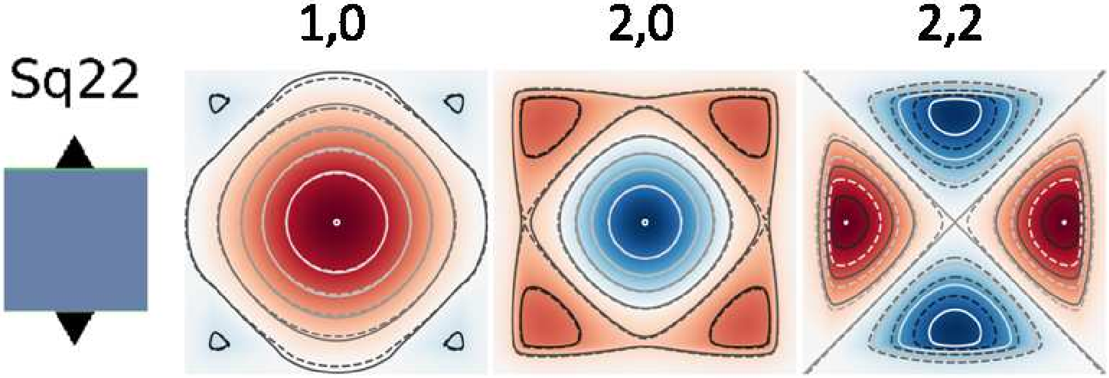

Figure S4: Iso-contour plots for the intensity of the near-fields for the RhoM-Sq04 (full lines) and RhoM-Sq22 (dashed lines) modes.

3 Mode 'elongation' for the RhoM-Sq22 nanocavity

The facets forming the RhoM-Sq04 and RhoM-Sq22 nanocavities are identical in size. The only differences are on the symmetry of the NP, with the RhoM-Sq04 having a four-fold symmetry whilw the RhoM-Sq22 a two-fold symmetry. In the main manuscript, we describe how the neighbouring triangular facets of the RhoM-Sq22 are at an anlge closer to the mirror, which allows for more charges to be accumulated at that edge. Therefore, the near-fields of the RhoM-Sq22 modes are different from the modes of RhoM-Sq04, even for the $m = 0$ modes that are centro-symmetric. In figure S4, we plot the near-field iso-contours for the intensity of the RhoM-Sq04 (full lines) and RhoM-Sq22 (dashed lines) modes, where one can see the differences between them. The largest impact is for the $(2, 2)$ mode, where the Ez-fields lose their zero value right at the centre. For the Sq04 nanocavity, the $E_z = 0$ iso-contours meet at the centre, while for the Sq22 nanocavity they avoid each other, which means that the E_z-field is non-zero at the centre. The 'elongation' effect can be seen more easily when comparing the RhOm-Sq22 nanocavity mode fields for two different edge curvatures. We show in figure S5 the $(2, 0)$ and $(2, 2)$ mode fields for edge curvatures of $\rho = 1$nm and $\rho = 5$nm, where the 'elongation' effect is more prominent.

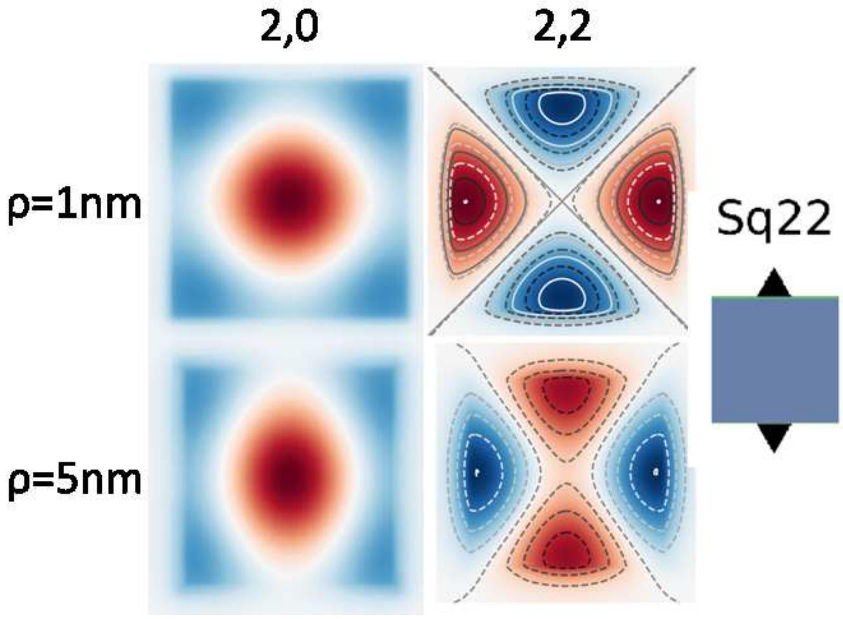

Figure S5: The near-fields of the $(2,0)$ and $(2,0)$ modes for the RhoM-Sq22 nanocavity, with edge curvature $\rho = 1$nm and $\rho = 5$nm.

4 Polyhedral Nanoantenna α-Coefficients

To complete the emitter transition frequencies selected with each facet assembly in Figure 5 of the main manuscript, here Figure S6 shows the α-coefficients for these same systems calculated for the reversed emitter frequencies. The same paths are considered, and the modal response of each system still in general follows the intensities of the corresponding near-field charge distributions.

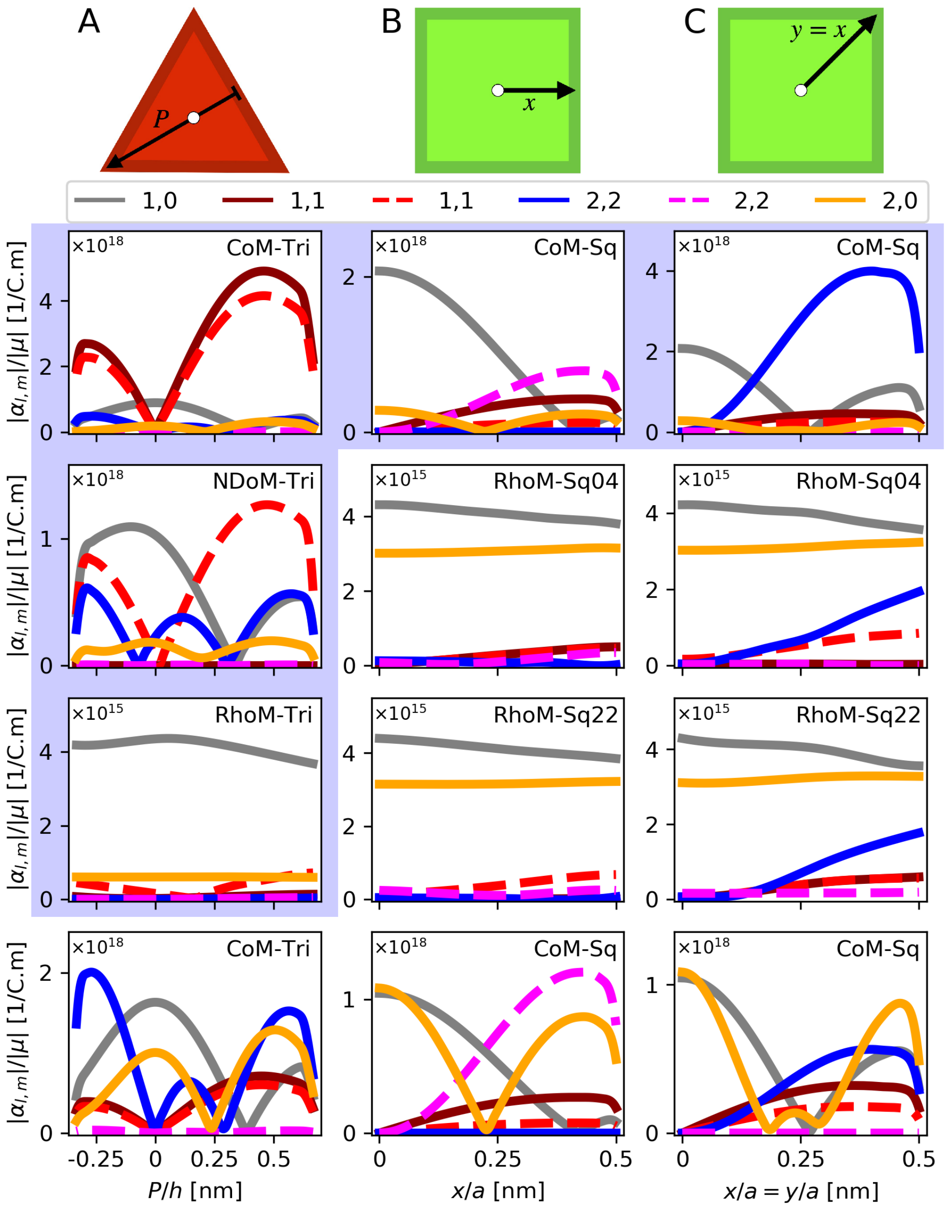

Figure S6: α-coefficients of the polyhedral NPoM geometries, for a series of emitter positions within the nanocavities. Column A - The triangular facets of the polyhedral systems, following a path from the facet edge to the opposing corner, through the centre of the facet—normalised to the height of the triangular facet ($h = \sqrt{3}a/2$). Column B - The square facets of the polyhedral systems, following a path along the x-axis from the centre of the facet. Column C - The square facets of the polyhedral systems, following a path along the diagonal from the centre of the facet. White and blue backgrounds respectively correspond to emitter transition wavelengths of $\lambda_{em} = 775$nm and $\lambda_{em} = 900$nm.

References

[1] Nuttawut Kongsuwan, Angela Demetriadou, Matthew Horton, Rohit Chikkaraddy, Jeremy J. Baumberg, and Ortwin Hess. Plasmonic Nanocavity Modes: From Near-Field to Far-Field Radiation. *ACS Photonics*, 7(2):463–471, 2020.

[2] Kalun Bedingfield, Eoin Elliott, Nuttawut Kongsuwan, Jeremy J. Baumberg, and Angela Demetriadou. Morphology dependence of nanoparticle-on-mirror geometries: A quasinormal mode analysis. *EPJ Appl. Metamat.*, 9:3, 2022.

[3] Jianji Yang, Jean-Paul Hugonin, and Philippe Lalanne. Light-in-complex-nanostructures/RETOP: Version 8.1, 2020.
